# Épidémiologie des troubles du sommeil en milieu rural au nord du Bénin en 2022

**DOI:** 10.11604/pamj.2025.51.60.47451

**Published:** 2025-06-01

**Authors:** Mendinatou Agbetou, Christian Tandjiekpon, Elfried Salanon, Oyéné Kossi, Hermionne Loko, Jennifer Mapaga Nyangui, Thierry Adoukonou

**Affiliations:** 1 Département de Neurologie, Université de Parakou, Parakou, Bénin,; 2 Unité d'Enseignement et de Recherche en Neurologie, Centre Hospitalier Universitaire Départemental du Borgou, Parakou, Bénin,; 3 Ecole Nationale de Formation des Techniciens Supérieurs en Santé Publique et en Surveillance Epidémiologique, Parakou, Bénin,; 4 Centre National Hospitalier Universitaire de Pneumo-phtisiologie de Cotonou, Cotonou, Bénin,; 5 Département de Neurologie, Université des Sciences de Santé de Libreville, Libreville, Gabon

**Keywords:** Troubles du sommeil, prévalence, qualité du sommeil, Bénin, Afrique, Sleep disorders, Prevalence, sleep quality, Bénin, Africa

## Abstract

**Introduction:**

les troubles du sommeil sont variés et de plus en plus fréquents chez les sujets africains, mais leur réelle prévalence est peu connue sur le continent. L´objectif de cette étude était de déterminer la prévalence des troubles du sommeil et les facteurs associés à la qualité du sommeil en milieu rural à Nikki au Bénin en 2022.

**Méthodes:**

il s'est agi d'une étude transversale à visée analytique réalisée chez les sujets âgés d'au moins 18 ans dans la commune de Nikki en 2022. La sélection des patients a été effectuée par sondage aléatoire à deux degrés. Les troubles du sommeil ont été diagnostiqués sur la base d'échelles validées telles le Pittsburg Sleep Quality Questionnaire, le Questionnaire de Berlin, le Score d'Epworth, le Restless Legs Syndrome Questionnaire, l'Index de Sévérité de l'Insomnie et le Munich Parasomnia Screening Questionnaire.

**Résultats:**

au terme de l'étude, 879 participants ont été inclus. Une prédominance féminine a été observée avec un sex ratio (F/H) de 1,9. La qualité du sommeil était mauvaise chez 52,21%. La prévalence de l'insomnie était de 9,44%, celle du syndrome des jambes sans repos de 21,39% et 21,16% pour la somnolence diurne. Un syndrome d'apnée du sommeil était présent chez 2,50% des participants. Le sexe masculin et le tabagisme actif étaient associés à la mauvaise qualité du sommeil avec respectivement p=0,03 et p=0,021.

**Conclusion:**

les troubles du sommeil sont fréquents en milieu rural à Nikki, nécessitant des campagnes de dépistage de masse pour en réduire la charge.

## Abstract (en)

**Introduction:**

sleep disorders are diverse and increasingly common among Africans, but their true prevalence is little known on the continent. The objective of this study was to determine the prevalence of sleep disorders and factors associated with sleep quality in rural areas of Nikki, Bénin, in 2022.

**Methods:**

this was an analytical cross-sectional study of subjects aged at least 18 years in the commune of Nikki in 2022. Patients were selected by two-stage random sampling. Sleep disorders were diagnosed on the basis of validated scales such as the Pittsburgh Sleep Quality Questionnaire, the Berlin Questionnaire, the Epworth Score, the Restless Legs Syndrome Questionnaire, the Insomnia Severity Index and the Munich Parasomnia Screening Questionnaire.

**Results:**

at the end of the study, 879 participants were included. Females predominated, with a sex ratio (F/H) of 1.9. Sleep quality was poor in 52.21% of cases. The prevalence of insomnia was 9.44%, restless leg syndrome 21.39% and daytime sleepiness 21.16%. Sleep apnea syndrome was present in 2.50% of participants. Male gender and active smoking were associated with poor sleep quality, with p=0.03 and p=0.021, respectively.

**Conclusion:**

sleep disorders are common in Nikki's rural area, requiring mass screening campaigns to reduce the burden.

## Introduction

La prévalence des troubles du sommeil est croissante en population générale allant de 20% à 50% selon les pays et les troubles recherchés [1-3]. L'Organisation mondiale de la santé (OMS) rapporte que la moitié de la population mondiale adulte risque de souffrir d'au moins un trouble du sommeil et il existe une association entre ces troubles du sommeil et les affections chroniques, les affections cardiovasculaires, les cancers [4]. Les sujets d'origine africaine ont tendance à avoir une durée de sommeil extrême et des troubles du sommeil plus fréquents que les caucasiens, avec comme conséquence l'élévation du risque de maladies cardiovasculaires dans cette population [5]. Malgré le nombre croissant d'études réalisées en Afrique, la médecine du sommeil y est peu développée et les données sur l'épidémiologie des troubles du sommeil sont insuffisantes [6]. L'insomnie, trouble du sommeil le plus fréquent, est en constante augmentation tant dans les pays occidentaux qu'africains [3]. Au Nigeria les auteurs rapportent que 44% des jeunes adolescents développent une insomnie [7]. La prévalence du syndrome d'apnée obstructive du sommeil varie de 56,7% au Maroc ou 57,7% au Cameroun, à 72,6% au Burkina Faso [8-10]. Le syndrome de jambes sans repos (SJSR) touche 3,5% de sujets dans une population de sujets âgés au Nigeria [11]. Au Bénin, l'étude BeSAS (Bénin Society and Sleep Study) qui a été menée en milieu urbain et rural au sud révèle que la prévalence du syndrome d'apnée-hypopnée du sommeil était de 57,51% [12]. En milieu urbain au nord, une prévalence de 39% de troubles du sommeil a été rapportée [3]. Cependant, les disparités mises en évidence dans l'étude BeSAS concernant la répartition des troubles du sommeil en milieu rural et urbain, ne permettent pas d'extrapoler les données disponibles actuellement sur les troubles du sommeil en milieu rural au nord du Bénin. Cette étude a été initiée avec comme objectif d'étudier la prévalence des troubles du sommeil et les facteurs associés à une mauvaise qualité du sommeil en milieu rural.

## Méthodes

La commune de Nikki a une superficie de 3171 km^2^ avec une population moyenne de 151232 habitants selon le quatrième Recensement Général de la Population et de l'Habitation réalisé en 2013. Elle est située dans le département du Borgou, à 529 km de Cotonou et à 115 km de Parakou. Elle compte trois cent cinquante-neuf (359) agglomérations regroupées en 54 villages appelés quartiers de ville répartis dans sept (7) arrondissements que sont Biro, Gnonkourokali, Ouénou, Nikki, Sérékali, Suya et Tasso. L'agriculture y est la principale activité professionnelle avec une superficie totale cultivée d'environ 65,8% de la superficie totale de la commune [13].

**Type et population d'étude:** il s'est agi d'une étude transversale à visée analytique conduite chez les sujets adultes âgés d'au moins 18 ans vivant dans la commune de Nikki depuis au moins 6 mois et ayant donné leur consentement libre et éclairé. Les participants répondant à ces critères d'inclusion mais incapables de répondre aux questions quel que soit le motif ont été exclus.

**Echantillonnage:** la taille de l'échantillon attendue a été calculée selon la formule de Schwartz à 571 sujets avec une prévalence de troubles du sommeil en milieu urbain à Parakou au nord du Bénin à 39% [3]. La sélection des patients a été effectuée par sondage aléatoire à deux degrés. La base de sondage était constituée par la liste des 54 quartiers de ville que comptaient les arrondissements de la commune de Nikki, avec le nombre respectif de leurs ménages. Au premier degré, un sondage aléatoire simple de 13% des 54 quartiers de ville a été effectué par tirage au sort à l'aide de la fonction formule « ALEA.ENTRE.BORNES. » soit sept (7) quartiers de ville. Au deuxième degré, la liste des sept quartiers de ville sélectionnés à la première étape était établie avec leur taille respective. Le nombre de participants à enquêter par quartier a été obtenu en divisant la taille de chaque quartier par la taille totale des sept quartiers multipliée par la taille calculée de l'échantillon. Dans chaque quartier, le premier ménage à enquêter était sélectionné par tirage aléatoire simple parmi la liste des ménages du quartier. Toutes les personnes remplissant les critères d'inclusion étaient enquêtées dans ce ménage, puis dans les ménages suivants de proche en proche jusqu'à hauteur du nombre de personnes à inclure par quartier.

**Variables:** la variable dépendante était la présence d'un trouble du sommeil. L'insomnie a été évaluée à l'aide de *l'Insomnia Severity Index*(ISI) avec un score supérieur à 8. Pour le SJSR, une réponse « Oui » aux quatre 1^res^ questions du *Restless Legs Syndrome Questionnaire* était considérée comme positive. Ces 4 critères ont une bonne sensibilité pour le diagnostic positif, soit 86% mais une spécificité de 45% améliorée à 90% lorsqu'un 5^e^ critère de diagnostic différentiel était appliqué [14,15]. Dans cette étude, le diagnostic était basé sur les 4 premiers critères. La sévérité a été évaluée à l'aide de *l'International Restless Legs Syndrome*. Tout patient ayant un score inférieur ou égal à 10 a été considéré comme ayant une atteinte légère, et celui qui avait un score compris entre 10 et 20 avait une atteinte modérée. Les scores compris entre 21 et 30 d'une part et entre 31 et 40 d'autre part ont été considérés comme étant respectivement une atteinte sévère et très sévère [14]. La somnolence diurne excessive a été évaluée à l'aide de l'échelle de somnolence d'Epworth pour tout patient ayant un score d'au moins 9. La présence d'apnée du sommeil a été définie par un score NoSAS d'au moins 8. La qualité du sommeil a été évaluée sur la base de l'échelle *Pittsburgh Sleep Quality Index* (PSQI). Un score inférieur ou égal à 5 définissait une bonne qualité de sommeil, cette dernière était mauvaise si le score était supérieur à 5.

Les variables indépendantes étaient socio-démographiques, environnementales, les facteurs comportementaux, les variables anthropométriques, les facteurs cliniques et paracliniques.

**Collecte des données:** les données ont été collectées dans une entrevue face à face avec un questionnaire prétesté, inspiré du questionnaire du Projet BeSAS et qui contenait l'ensemble des échelles validées sur les troubles du sommeil [12]. L'équipe de collecte a été préformé sur le recueil des données. Les mesures physiques ont été prises à l'aide d'appareils répondant aux normes de l'OMS, conformément aux procédures recommandées. Le poids était mesuré avec un pèse-personne portable, étalonné avant usage, de type électronique de marque Omron: poids minimal 200 grammes (g), poids maximal 180 kilogrammes (kg), ayant une précision de 100 g. La taille était mesurée en centimètre (cm) par une toise adulte mobile de marque SECA en position debout sans port de chaussures ou relevée par les pièces administratives identitaires (cartes nationales, passeport). L'indice de masse corporelle (IMC) était calculé par le rapport du poids en kilogramme sur la taille en mètre carré et était exprimé en kilogramme par mètre carré (kg/m^2^). Entre 18,5 et 24,9, l'IMC est normal. De 25 à 29,9 il s'agit d'un surpoids, de 30 à 34,9 une obésité modérée, de 35 à 39,9 une obésité sévère, supérieur ou égal à 40 une obésité morbide. La tension artérielle a été mesurée le jour de la collecte en position assise, sur le bras droit, après que le participant se soit reposé pendant 15 minutes, à l'aide d'un tensiomètre électronique OMRON^®^muni d'un brassard. Trois mesures ont été prises à 5 minutes d'intervalle; la moyenne des deux dernières mesures a été utilisée pour définir la tension artérielle. Une tension artérielle supérieure à 140/90 mmHg était considérée comme élevée. La glycémie capillaire à jeun a été mesurée avec un glucomètre de marque Accu-Chek^®^en g/L. Une valeur entre 0,7 et 1,1g/l était considérée comme normale.

**Analyse des données:** les données ont été analysées avec le logiciel R 4.4.1. Les paramètres de tendance centrale et de dispersion ont été estimés pour les variables quantitatives et les proportions associées à leur intervalle de confiance (IC) à 95% pour les variables qualitatives. Une régression logistique binaire multiple a été utilisée en analyse multivariée pour ressortir les facteurs associés à la qualité du sommeil. Pour étudier le sens des associations, les rapports de prévalence et leurs intervalles de confiance à 95% ont été calculés. Le seuil de significativité était inférieur à 0,05.

**Considération d'ordre éthique:** le protocole de ce travail de recherche a été soumis au comité local d'éthique de l'Université de Parakou (CLERB-UP). L'anonymat et la confidentialité des informations collectées ont été respectés.

## Résultats

**Caractéristiques générales:** au terme de cette étude, 879 sujets d'âge moyen de 35,32 ± 15 ans avec des extrêmes allant de 18 à 98 ans ont été inclus. On notait une prédominance féminine avec un sex ratio (F/H) de 1,9. Plus de la moitié (59,3%) des personnes enquêtées n'avait aucune instruction officielle. La plupart étaient mariés et 91,6% exerçaient une profession indépendante (Tableau 1). Cent trente-sept sujets, soit 15,9% avaient un antécédent de tabagisme, dont 11,1% actif; 4,9% consommait régulièrement de l'alcool; 65,5% ne pratiquaient pas d'activité physique régulière et 2,8% consommaient régulièrement du café. Les antécédents d'hypertension artérielle et de diabète étaient présents chez respectivement 9,8% et 4,9% de sujets (Tableau 2). La tension artérielle était élevée le jour de l'enquête chez 148 sujets et 14 sujets avaient une glycémie élevée (Tableau 3).

**Tableau 1:** caractéristiques socio-démographiques et environnementales (N=879; Nikki 2022)

		Qualité sommeil					
		Bonne (n=420)		Mauvaise (n=459)	RP	IC95%	p
**Tranche d'âge**							
[18-20[		23 (37,1%)		39 (62,9%)	1	-	0,436
[20-30[		166 (49,4%)		170 (50,6%)	0,6	0,34-1,04	0,076
[30-40[		95 (50,5%)		93 (49,5%)	0,57	0,31-1,03	0,067
[40-50[		61 (48,4%)		65 (51,6%)	0,62	0,33-1,16	0,143
[50-60[		35 (42,2%)		48 (57,8%)	0,8	0,40-1,58	0,537
≥60		40 (47,6%)		44 (52,4%)	0,64	0,32-1,26	0,205
**Sexe**							
Féminin		290 (50,4%)		285 (49,6%)	1	-	-
Masculin		130 (42,8%)		174 (57,2%)	1,36	1,03-1,80	**0,03**
**Niveau d'instruction**							0,404
Aucun		259 (49,7%)		262 (50,3%)	1,41	0,44-4,83	0,556
Primaire		91 (43,8%)		117 (56,3%)	1,8	0,55-6,25	0,328
Secondaire		63 (45,7%)		75 (54,3%)	1,66	0,50-5,87	0,402
Supérieur		7 (58,3%)		5 (41,7%)	1	-	0,374
**Statut matrimonial**							
Célibataire		61 (41,5%)		86 (58,5%)	1	-	0,324
Divorcé		7 (46,7%)		8 (53,3%)	0,81	0,27-2,42	0,699
Marié(e)		334 (49,3%)		343 (50,7%)	0,72	0,50-1,04	0,085
Veuf(ve)		18 (45%)		22 (55%)	0,86	0,42-1,76	0,69
**Profession**							
Bénévole		8 (57,1%)		6 (42,9%)	1		
Employé		6 (54,5%)		5 (45,5%)	1,11	0,22-5,57	0,896
Etudiant		7 (28%)		18 (72%)	3,42	0,88-14,27	0,078
Indépendant		390 (48,4%)		416 (51,6%)	1,42	0,49-4,35	0,517
Invalide		8 (42,1%)		11 (57,9%)	1,83	0,45-7,71	0,394
Retraite		1 (25%)		3 (75%)	4	0,39-92,85	0,276
**Nuisance nocturne**							
Non		308 (48,7%)		325 (51,3%)	1		
Oui		112 (45,5%)		134 (54,5%)	1,13	0,84-1,52	0,404
**Utilisation de téléphone portable au coucher**							
Non	156 (51,8%)		145 (48,2%)		1		
Oui	264 (45,7%)		314 (54,3%)		1,27	0,96-1,69	0,083
**Chambre à proximité de la route**							
Non	313 (47,7%)		343 (52,3%)		1		
Oui	107 (48%)		116 (52%)		0,98	0,72-1,34	0,944
							

**Tableau 2:** antécédents et mode de vie (N=879; Nikki 2022)

	Qualité sommeil				
	Bonne (n=420)	Mauvaise (n=459)	RP	IC95%	p
**Tabac**					
N'a jamais fumé	365 (49,2%)	377 (50,8%)	1	-	0,254
Fume actuellement	36 (36,7%)	62 (63,3%)	1,66	1,08-2,59	0,021
Ne fume plus	19 (48,7%)	20 (51,3%)	1,01	0,53-1,95	0,954
**Café**					
Jamais	358 (49%)	373 (51%)	1	-	-
Occasionnellement	53 (43,1%)	70 (56,9%)	1,26	0,86-1,86	0,227
Régulièrement	9 (36%)	16 (64%)	1,7	0,75-4,07	0,206
**Consommation Thé**					
Non	308 (47,2%)	345 (52,8%)	1		0,807
Oui	112 (49,6%)	114 (50,4%)	0,9	0,67-1,23	0,535
**Alcool**					
Jamais	345 (49,1%)	357 (50,9%)	1	-	0,144
Occasionnellement	60 (44,8%)	74 (55,2%)	1,19	0,82-1,73	0,354
Régulièrement	15 (34,9%)	28 (65,1%)	1,8	0,96-3,51	0,072
**Activité physique**					
Intensif	31 (43,7%)	40 (56,3%)	1	-	-
Jamais	273 (47,4%)	303 (52,6%)	0,86	0,52-1,41	0,552
Occasionnel	56 (50%)	56 (50%)	0,77	0,42-1,40	0,403
Régulier	60 (50%)	60 (50%)	0,77	0,42-1,39	0,397
ATCD* HTA					
Non	384 (48,4%)	409 (51,6%)	1	-	-
Oui	36 (41,9%)	50 (58,1%)	1,3	0,83-2,05	0,248
ATCD* Diabète					
Non	405 (48,4%)	431 (51,6%)	1	-	-
Oui	15 (34,9%)	28 (65,1%)	1,75	0,93-3,41	0,086
ATCD* Asthme					
Non	386 (48,6%)	408 (51,4%)	1	-	-
Oui	34 (40%)	51 (60%)	1,41	0,90-2,25	0,132

**Tableau 3:** constantes cliniques et paraclinique (N=879; Nikki 2022)

	Qualité sommeil				
	Bonne (n=420)	Mauvaise (n=459)	RP	IC95%	p
**Tension artérielle**					
Normale	354 (48,4%)	377 (51,6%)	1		0,395
Elevée	66 (44,6%)	82 (55,4%)	1,16	0,81-1,66	0,966
**IMC**					
Poids normal	285 (47,6%)	314 (52,4%)	1	-	0,624
Dénutrition	42 (46,2%)	49 (53,8%)	1,05	0,68-1,65	0,799
Surpoids	71 (49,3%)	73 (50,7%)	0,93	0,64-1,34	0,709
Obésité	22 (48,9%)	23 (51,1%)	0,94	0,51-1,74	0,865
**Glycémie**					
Normale	284 (48,9%)	297 (51,1%)	1	-	-
Hypoglycémie	129 (45,4%)	155 (54,6%)	1,14	0,86-1,52	0,339
Hyperglycémie	7 (50%)	7 (50%)	0,95	0,32-2,82	0,934

**Prévalence des troubles du sommeil:** au total, 495 sujets avaient rapporté des troubles du sommeil, soit une prévalence globale de troubles du sommeil de 52,21%. Les principaux troubles du sommeil dépistés étaient le SJSR (21,39%), la somnolence diurne excessive (21,16%), l'insomnie (9,44%) et le syndrome d'apnée/hypopnée du sommeil (2,50%). Sur les 83 sujet souffrant d'insomnie, respectivement 66, 15 et 2 sujets avaient une insomnie légère, modérée et sévère. Quant au SJSR 79 étaient légers (8,99%), 84 modérés (9,56%), 20 sévères (2,28%) et 5 très sévères (0,57%).

**Facteurs associés à la mauvaise qualité du sommeil:** la qualité du sommeil était mauvaise chez 459 sujets, soit 52,22%. La moyenne d'heures de sommeil par nuit était de 4,1±1,49 heures avec des extrêmes allant de 2h à 12h. Les facteurs associés à la mauvaise qualité du sommeil en analyse bivariée étaient le sexe masculin RP 1,36 IC [1,03-1,80] valeur de p= 0,03 et le tabagisme actif RP 1,66 IC [1,08-2,59] Valeur de p= 0,021. (Tableau 1, Tableau 2).

## Discussion

Au terme de cette étude, la prévalence de troubles du sommeil était de 52,21%. Les principaux types de troubles du sommeil étaient le SJSR (21,39%), la somnolence diurne excessive (21,16%), l'insomnie (9,44%) et le syndrome d'apnée/hypopnée du sommeil (2,50%). Les facteurs associés à la qualité du sommeil étaient le sexe, le tabagisme actif. Les troubles du sommeil font le lit des maladies cardiovasculaires en raison des perturbations de l'axe hypothalamo-hypophyso-surrénalien et du dysfonctionnement du système autonome [16]. En effet, un sommeil court ou long est associé à une obésité accrue, un diabète, l'hypertension et les maladies cardiovasculaires [17]. Contrairement à une récente étude menée à Parakou en milieu urbain où le premier trouble du sommeil rapporté était l'insomnie (21,6%) [3], le SJSR est le trouble du sommeil le plus fréquent en milieu rural avec une prévalence élevée dans cette étude. La plupart des études africaines rapportent une prévalence plus faible que chez les sujets caucasiens. En milieu urbain au nord du Bénin comme chez les sujets âgés au Nigéria ou encore en population urbaine en Tanzanie, la prévalence du SJSR varie de 0,037% à 6% [3,11,18]. Même si le SJSR est le symptôme le plus courant chez les sujets caucasiens, sa prévalence dépasse rarement 15% surtout dans des populations spécifiques comme chez les sujets souffrant de sclérose en plaques où l'on rapporte 41,3% de fréquence de SJSR [19-21]. Cette prévalence varie en fonction des tranches d'âge allant de 3% chez les 18-29 ans à 19% chez les plus de 80 ans et seul 2,5% présentent des formes sévères nécessitant un traitement [22,23]. Elle est élevée dans notre série en rapport d'une part avec l'outil diagnostique utilisé car les 4 questions du *Restless Legs Syndrome Questionnaire* sont de bonne sensibilité mais restent peu spécifiques. Le 5^e^ critère de confirmation basé sur l'élimination des diagnostics différentiels n'ayant pas été appliqué dans cette étude, le nombre réel de cas de SJSR reste à confirmer. D'autre part, la suggestivité des réponses des sujets enquêtés pourrait avoir surévalué la fréquence de cette pathologie et donc être responsable d'un biais d'information. Aussi nous pourrions ici être confrontés à un taux élevé de faux positifs, comme souligné chez les patients souffrant de sclérose en plaque [24]. Enfin cette prévalence élevée pourrait aussi être liée au fait que le SJSR est un trouble sensitivo-moteur ayant un impact profond sur le sommeil [25] comme l'ensemble des troubles sensitivo-moteurs liés à d'autres affections telles que les atteintes du système nerveux périphérique fréquentes dans cette population en raison des caractéristiques socio-professionnelles et économiques des sujets enquêtés. Dans cette étude, la prévalence de l'insomnie en population générale était de 9,44%. Ce résultat est inférieur au 30,7% rapporté au Nigéria, où l'insomnie a été évaluée à l'aide du questionnaire de l'OMS Composite International Diagnostic Interview, version 3 (CIDI) [26]. Le type de questionnaire utilisé pourrait expliquer cette différence observée. En effet, en matière de dépistage de l'insomnie, le questionnaire le mieux adapté est celui de l'Index de Sévérité de l'Insomnie. Par ailleurs, il présente une spécificité plus importante que le CIDI justifiant la prévalence plus faible observée. Ce résultat est également faible par rapport à celui rapporté à Parakou (22%) [3]. Ici les conditions de vie et de nuisance du milieu urbain, de même que la généralisation de l'usage du téléphone au coucher, le stress, l'anxiété, les problèmes personnels non résolus, la dégradation du pouvoir d'achat, le bruit sont autant de facteurs pouvant justifier cette prévalence plus élevée en milieu urbain comparé au milieu rural. En effet, il est prouvé que la pollution sonore, la pollution de l'air, l'environnement bâti et l'existence ou non d'espace vert interfèrent avec la capacité à s'endormir, à rester endormi et à se réveiller reposé [27]. La prévalence de la Somnolence diurne excessive était de 21,16%. La prévalence de la somnolence diurne excessive dans notre étude est élevée comparée à celle rapportée en milieu urbain à Parakou (15%) [3] comme dans le *Middle East and North Africa* (MENA) où l'hypersomnolence est le trouble du sommeil le plus fréquent dans une étude réalisée chez les étudiants en médecine [28]. Nous pouvons justifier cette différence dans notre étude par l'état de l'environnement qui est calme et sans bruit en milieu rural, favorisant ainsi une augmentation de l'envie de sommeil pendant la journée. La fatigue entrainée par les travaux champêtres peut parfois occasionner des nuits sans sommeils et peut augmenter le besoin de sommeil dans la journée également. La prévalence du SAOS était de 72,6% au Burkina Faso [10] bien plus élevée que les 2,5% notés dans cette étude pour le syndrome d'apnée-hypopnée du sommeil. En effet, ici nous n'avons pas réalisé d'enregistrement polygraphique. Ce qui pourrait sous-estimer le nombre de cas dans notre série. Notre résultat est proche de celui trouvé par d'autres auteurs, allant de 2,1% à 3,3% [29,30]. La forte suspicion de syndrome d'apnée du sommeil a été rapportée dans 9,6% des cas dans une population africaine au Burkina Faso [10] et constitue un facteur de risque d'hypertension artérielle [31]. Dans l'étude BeSAS, les troubles respiratoires du sommeil sont plus fréquents chez les participants vivant en zones urbaines par rapport aux zones rurales, avec une prédominance des troubles légers à sévères (index apnée-hypopnée ≥5/h) chez environ 43% [12]. Comme attendu, cette prévalence est faible et liée au milieu de vie rural en raison de la réduction de la pollution et de l'obésité [32-34]. Le sexe masculin était significativement associé et multipliait par 1,36 le risque d'avoir une mauvaise qualité de sommeil dans cette étude. Ce résultat est le même que celui rapporté dans l'étude BeSAS [12]. Par contre, plusieurs auteurs ont rapporté que les femmes sont plus susceptibles de se plaindre de l'insatisfaction du sommeil que les hommes [35]. La mauvaise qualité du sommeil chez les jeunes adolescents scolarisés était de 12,2% au Nigeria [36]. Elle est un des facteurs de risque du syndrome d'apnée du sommeil tout autant que l'obésité [10]. Le tabagisme actif était associé à la mauvaise qualité du sommeil dans cette étude. Il est démontré que le tabagisme induit un sommeil trop court de mauvaise qualité ou un manque de sommeil avec une somnolence diurne importante [37-39]. C'est un facteur de risque indépendant de dépression, de stress, d'anxiété et de mauvaise qualité du sommeil chez les jeunes adultes [40]. Ceci souligne l'importance des mesures de lutte contre le tabagisme.

**Les limites:** l'outil diagnostic étant basé sur un questionnaire, les biais d'information ont pu être majorés. La non-utilisation de la polysomnographie a pour conséquence un manque de précision dans les diagnostics et peut donc avoir surévalué la prévalence des troubles. Ces limites peuvent relativiser la généralisation des résultats de cette étude à l'ensemble de la population.

## Conclusion

Cette étude de dépistage révèle une prévalence élevée des troubles du sommeil et une mauvaise qualité du sommeil dans la population rurale de Nikki. Il urge de dépister ces troubles du sommeil à grande échelle en vue d'une prévention globale des facteurs de risque vasculaire et d'une réduction de morbi-mortalité élevée des maladies non transmissibles.

### Etat des connaissances sur le sujet

- L'insomnie est le trouble du sommeil le plus fréquent en milieu urbain au nord du Bénin avec une prévalence de 22%;
- La prévalence de la somnolence diurne excessive est de 15% et le sommeil est de mauvaise qualité chez 39% en milieu urbain au nord du Bénin;
- La prévalence du syndrome d'apnée-hypopnée du sommeil léger à sévère est de 43,6%; modéré à sévère de 11,6% et sévère de 2,7% au sud du Bénin.

### Contribution de notre étude à la connaissance

- La prévalence du syndrome des jambes sans repos est élevée en milieu rural au nord du Bénin et celle de l'insomnie est plus faible;
- Les facteurs associés à la mauvaise qualité du sommeil sont le sexe masculin et le tabagisme actif.
